# Supplementary material for: Chemistry and Bioactivity of Croton Essential Oils: Literature Survey and Croton hirtus from Vietnam
Source: Molecules. 2023 Mar 3;28(5):2361. doi: 10.3390/molecules28052361 (PMC10005233; doi:10.3390/molecules28052361)
Supplement: Supplementary file 1 [file molecules-28-02361-s001.zip › molecules-2212585-supplementary.pdf]

Table S1. Chemical composition of *Croton hirtus* essential oil.

RI	Compound name	Area %
932	α-Pinene	tr
948	Camphene	tr
971	Sabinene	0.1
977	β-Pinene	tr
987	Myrcene	tr
1023	*p*-Cymene	0.1
1028	Limonene	0.1
1031	1,8-Cineole	0.1
1033	(*Z*)-β-Ocimene	tr
1044	(*E*)-β-Ocimene	tr
1098	Linalool	tr
1101	Hotrienol	tr
1103	Nonanal	tr
1111	4,8-Dimethylnona-1,3,7-triene	0.1
1283	Bornyl acetate	0.1
1330	δ-Elemene	0.1
1331	Bicycloax-4(15)-ene	0.1
1345	α-Cubebene	0.2
1356	Neryl acetate	0.1
1368	Cyclosativene	0.7
1375	α-Copaene	2.9
1383	β-Bourbonene	3.1
1386	β-Cubebene	0.8
1389	β-Elemene	9.1
1402	α-Gurjunene	2.6
1422	β-Caryophyllene	32.8
1427	γ-Elemene	0.5
1429	β-Copaene	0.5
1434	α-Guaiene	0.2
1437	Aromadendrene	0.1
1443	Isogermacrene D	0.3
1452	1,2,2α,3,3,4,6,7,8,8α-Decahydro-2α,7,8-trimethylacenaphthylene *	0.5
1456	α-Humulene	8.5
1459	*allo*-Aromadendrene	0.9
1467	*cis*-Muurola-4(14),5-diene	0.2
1472	α-Helmiscapene	0.5
1474	γ-Muurolene	1.3
1481	Germacrene D	11.6
1483	γ-Gurjunene	0.6
1488	β-Selinene	0.6

1491	γ-Amorphene	0.9
1494	Bicyclogermacrene	1.5
1496	α-Muurolene	1.0
1501	(*E*,*E*)-α-Farnesene	0.2
1506	Geramcrene A	0.1
1511	γ-Cadinene	0.3
1513	Cubebol	0.2
1516	δ-Cadinene	1.4
1518	7-*epi*-α-Selinene	0.3
1519	*trans*-Calamenene	0.1
1535	α-Cadinene	0.2
1540	*cis*-Calamenene	tr
1550	Isocaryphyllene oxide	0.3
1557	Germacrene B	1.2
1575	Spathulenol	0.8
1581	Caryophyllene oxide	5.0
1584	Globulol	0.1
1608	Humulene epoxide II	0.8
1624	Selin-6-en-4β-ol	0.3
1626	1-*epi*-Cubenol	0.1
1635	Caryophylla-4(12),8(13)-dien-5β-ol	0.2
1637	*allo*-Aromadendrene epoxide	0.4
1640	τ-Cadinol	0.3
1642	τ-Muurolol	0.2
1644	α-Muurolol (Torreyol)	0.1
1650	Pogostol	0.2
1653	α-Cadinol	1.1
1656	Selin-11-en-4α-ol	0.2
1941	*iso*-Phytol	0.1
2102	Phytol	0.4
2207	Phytyl acetate	0.1
	Monoterpene hydrocarbons	0.2
	Oxygenated monoterpenoids	0.2
	Sesquiterpene hydrocarbons	85.7
	Oxygenated sesquiterpenoids	9.7
	Diterpenoids	0.7
	Others	0.1
	Total Identified	96.6

* This compound is in the *Dictionary of Natural Products*, but there is no trivial name. tr: trace.
